# RnaSeqSampleSize: real data based sample size estimation for RNA sequencing

**DOI:** 10.1186/s12859-018-2191-5

**Published:** 2018-05-30

**Authors:** Shilin Zhao, Chung-I Li, Yan Guo, Quanhu Sheng, Yu Shyr

**Affiliations:** 1 Department of Biostatistics, Vanderbilt University Medical Center, Nashville, TN 37232 USA; 2 Department of Statistics, National Cheng Kung University, Tainan, 70101 Taiwan; 3 Department of Internal Medicine, University of New Mexico, Albuquerque, NM 87131 USA; 4 Key Laboratory of Resource Biology and Biotechnology in Western China, School of Life Sciences, Northwest University, Xi’an, 710069 Shanxi China

**Keywords:** RNA-Seq, Sample size, Power analysis, Simulation

## Abstract

**Background:**

One of the most important and often neglected components of a successful RNA sequencing (RNA-Seq) experiment is sample size estimation. A few negative binomial model-based methods have been developed to estimate sample size based on the parameters of a single gene. However, thousands of genes are quantified and tested for differential expression simultaneously in RNA-Seq experiments. Thus, additional issues should be carefully addressed, including the false discovery rate for multiple statistic tests, widely distributed read counts and dispersions for different genes.

**Results:**

To solve these issues, we developed a sample size and power estimation method named RnaSeqSampleSize, based on the distributions of gene average read counts and dispersions estimated from real RNA-seq data. Datasets from previous, similar experiments such as the Cancer Genome Atlas (TCGA) can be used as a point of reference. Read counts and their dispersions were estimated from the reference’s distribution; using that information, we estimated and summarized the power and sample size. RnaSeqSampleSize is implemented in R language and can be installed from Bioconductor website. A user friendly web graphic interface is provided at http://cqs.mc.vanderbilt.edu/shiny/RnaSeqSampleSize/.

**Conclusions:**

RnaSeqSampleSize provides a convenient and powerful way for power and sample size estimation for an RNAseq experiment. It is also equipped with several unique features, including estimation for interested genes or pathway, power curve visualization, and parameter optimization.

**Electronic supplementary material:**

The online version of this article (10.1186/s12859-018-2191-5) contains supplementary material, which is available to authorized users.

## Background

RNA sequencing is a powerful NGS tool that has been widely used in differential gene expression studies [1]. One of the most important steps in designing an RNA sequencing experiment is selecting the optimal number of biological replicates to achieve a desired statistical power (sample size estimation), or estimating the likelihood of successfully finding the statistical significance in the dataset (power estimation). An insufficient number of replicates may lead to unreliable conclusions, whereas too many replicates may result in a waste of time and resources. The tradeoff between cost and study power needs to be carefully balanced. To address this issue, several attempts have been made to estimate power and sample size for RNA-seq experiments.

Sample size and power analysis have been well-established for traditional biological studies such as genome wide association studies (GWAS) and microarray gene expression studies [2, 3]. In earlier RNA-Seq studies, the analysis was based on Poisson distribution, because RNA-Seq data can be represented as read counts [4–6]. It was discovered, however, that Poisson distribution does not fit the empirical data due to an over-dispersion mainly caused by natural biological variation [7, 8]. To address this issue, a few negative binomial distribution-based methods have been developed. These methods provide researchers with more flexibility in assigning between-sample variations [9–13]. Hart et al. [14] proposed a power analysis method based on the score test for single-gene differential expression analysis. This method has been implemented in Bioconductor as RNASeqPower. To handle multiple gene comparisons, Li et al. [15] proposed a power analysis method while controlling for the false discovery rate. To incorporate the experiment’s budget into the power analysis, Wu et al. [16] introduced the concepts of stratified power by coverage or biological variation and the cost of false discovery, then proposed a simulation-based method for power analysis. The method was implemented as a Bioconductor package PROPER.

However, there are several limitations in the majority of the previous methods, such as: the lack of properly accounting for average read counts and dispersion in different genes; the lack of proper reference data; and the lack of easy and user friendly interfaces. The average read counts of genes are distributed in a range of more than four orders of magnitude, and their dispersions are highly dependent on their gene expression level [17, 18]. Previous estimation methods were not designed for these distributions, so they often utilized one value chosen conservatively or by experience [9, 10], which often resulted in an over-estimated sample size. Yu et al. [18] have introduced a simulation-based procedure which considers dependence between gene expression level and its dispersion, but this method has not been made into an easy-to-use software. Additionally, it is computationally expensive to apply these methods to every gene in the dataset, because the individual power analysis for the exact test involves infinite sums, and the study’s overall power is estimated from a summation of the individual power. A proper approach is to providing reference data with similar distributions to current experiments. We acknowledge that such data may not be available for every project type and that a significant amount of programming and data processing effort is needed to utilize them.

To address the aforementioned problems, we used previous methods [15] as the foundation for developing RnaSeqSampleSize package, which controls the false discovery rate (FDR) of multiple testing, and utilizes the average read count and dispersion distributions from real data to estimate a more reliable sample size. The package is also optimized for running efficiency and provides additional features, which we demonstrate using real RNA-Seq data.

## Results and discussion

**Fig. 1:**
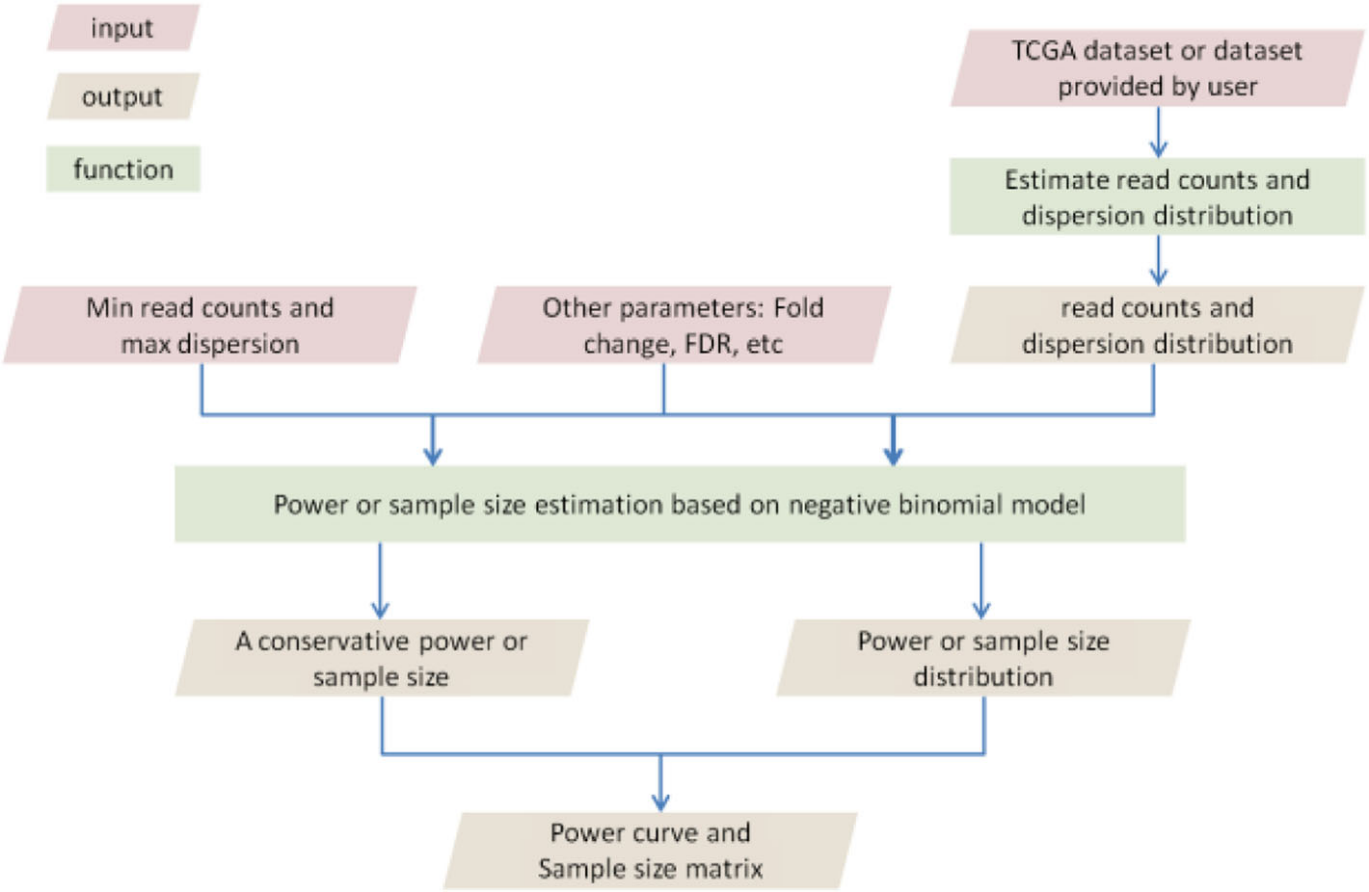
RnaSeqSampleSize package workflow

The detailed feature list of RnaSeqSampleSize package can be observed in Fig. 1:

### Sample size estimation with single average read count and dispersion

RnaSeqSampleSize was developed based on the sample size and power estimation methods described in the previous study [10], and it greatly improved the compatibility and efficiency of older methods. In this new implementation, a minimal average read count and a maximal dispersion are used to represent all genes in the RNA sequence experiments, and a conservative sample size or power can be estimated. More importantly, RnaSeqSampleSize is compatible for large average read counts and dispersions, supporting as much as a 2000 average read count. We optimized the running efficiency of the method from 40 min to two seconds for most of the widely used parameters (Additional file 1: Table S1).

### Sample size estimation with real data

**Fig. 2:**
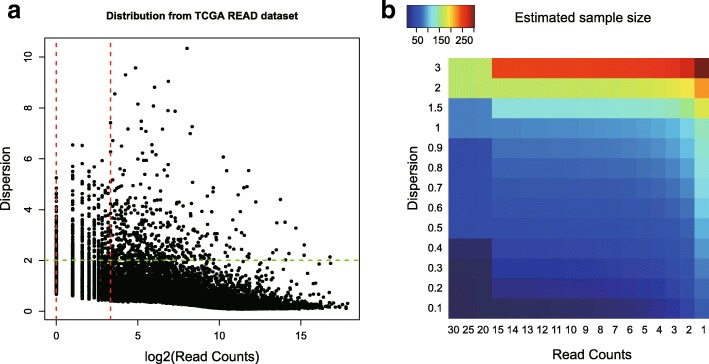
Read counts and dispersion distribution greatly influence the estimated sample size and power. **a** The read counts and dispersion distribution for all genes from TCGA Rectum adenocarcinoma (READ) dataset. The red lines indicate read counts equal to one and 10. And the green line indicates the 95% quantile of all gene dispersions. **b** The estimated sample size required to achieve 0.8 power in different combinations of read counts and dispersions

As previously stated, average read counts and dispersions for genes have wide distributions within a single RNA sequencing experiment. A tiny fluctuation in the average read count or dispersion will greatly influence the estimated power or sample size (Fig. 2). For example, in TCGA Rectum adenocarcinoma (READ) dataset, the genes have a dispersion from 0 to 10, and the average read counts range from one to numbers in the several thousands (Fig. 2a). In such a scenario, the sample size estimation from a single value is inaccurate. We computed that the estimated sample size increased from 10 to 302 when the minimal average read count changed from one to 30 and the maximal dispersion changed from 0.1 to three (Fig. 2b).

Instead of relying on guessing to discover the future data’s distribution, RnaSeqSampleSize uses data parameters from previous studies. To demonstrate this feature, we compared the usual sample size estimation approach to RnaSeqSampleSize’s empirical approach. We used the datasets from TCGA as the reference data to estimate the true data distribution. TCGA is a cancer consortium data set considered to be the most representative dataset for cancer RNA-seq. Following common standards, we set the minimum average read count as one or 10, and 95% quantile in all gene dispersions as maximum dispersion, while the empirical databased method utilized the empirical average read count and dispersion distribution computed from TCGA (Additional file 1: Table S2 and S3). The estimated sample size obtained using the empirical data-based method was smaller in all parameter combinations (Additional file 1: Table S2 and S3). For example, the estimated sample size for TCGA Rectum adenocarcinoma (READ) dataset was 168 when the following parameters were used: fold change at 2; desired power at 0.8; FDR at0.05; minimal read count at 10; dispersion at 2.0 (Additional file 1: Table S2, in boldface). This estimated sample size is larger than the real sample size, because we used the lowest read count and highest dispersion to represent all genes, even if most of them were not very conservative (Red line and green line in Fig. 2a).

**Fig. 3:**
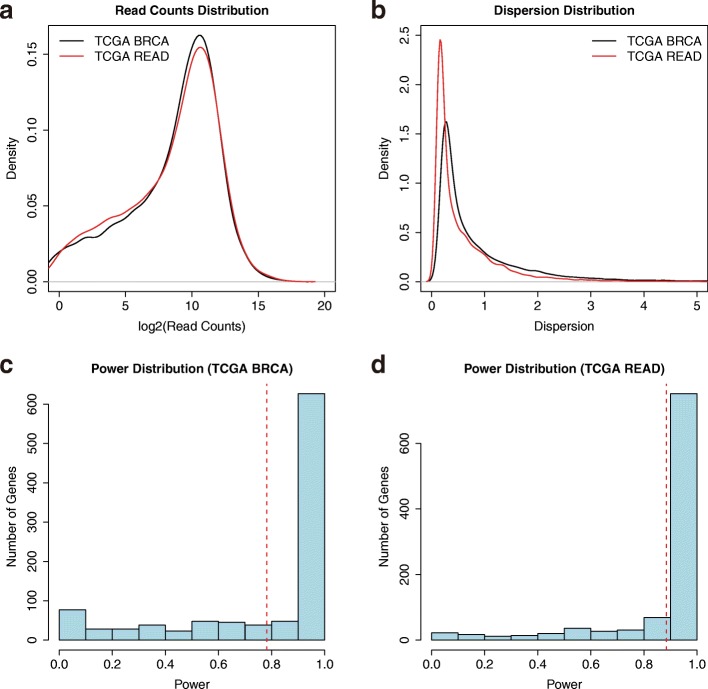
Sample size estimation with real data. **a** The read counts distribution for all genes from TCGA Breast Invasive Carcinoma (BRCA) and Rectum adenocarcinoma (READ) dataset; (**b**) The dispersion distribution for all genes from TCGA BRCA and READ dataset; (**c**) The power distribution based on the count and dispersion distributions in TCGA BRCA dataset when sample size equals 71. The red lines indicate the mean value of power distribution. **d** The power distribution based on the count and dispersion distributions in TCGA READ dataset when sample size equals 71. The red lines indicate the mean value of power distribution

In the empirical data-based method, the genes in the reference dataset were randomly selected, and the powers were estimated respectively based on these genes (Fig. 3). With the same desired power and FDR, the estimated sample size was 42 (Additional file 1: Table S3, in boldface). This result is a better representation of the genes in RNA-seq experiment and it is substantially less than the result that was obtained using the traditional method. More importantly, the empirical data-based method can reflect the differential gene expression pattern in a different dataset. For example, genes in TCGA Breast Invasive Carcinoma (BRCA) and READ dataset have similar read count distributions (Fig. 3a), but genes in BRCA dataset have a higher dispersion than in READ (Fig. 3b). Thus, when analyzing genes in READ dataset, we have a higher power distribution (Fig. 3c and d), suggesting that less samples are needed to analyze rectum adenocarcinoma samples with a desired power.

### Sample size estimation for interested genes or pathways

In certain situations, researchers may be interested in a subset of genes defined by certain features such as shared pathways or gene ontology categories, rather than the entire gene set. In such scenarios, the sample size estimation method needs to be adjusted, because the gene subsets of interest may have distinct expression patterns compared to other genes [19]. RnaSeqSampleSize was designed to handle sample size and power analysis in such experiment design by allowing users to provide a list of interested genes or a KEGG pathway ID; this ensures that only the read count and dispersion distribution of interested genes or genes in the selected pathway will be used for estimation.

**Fig. 4:**
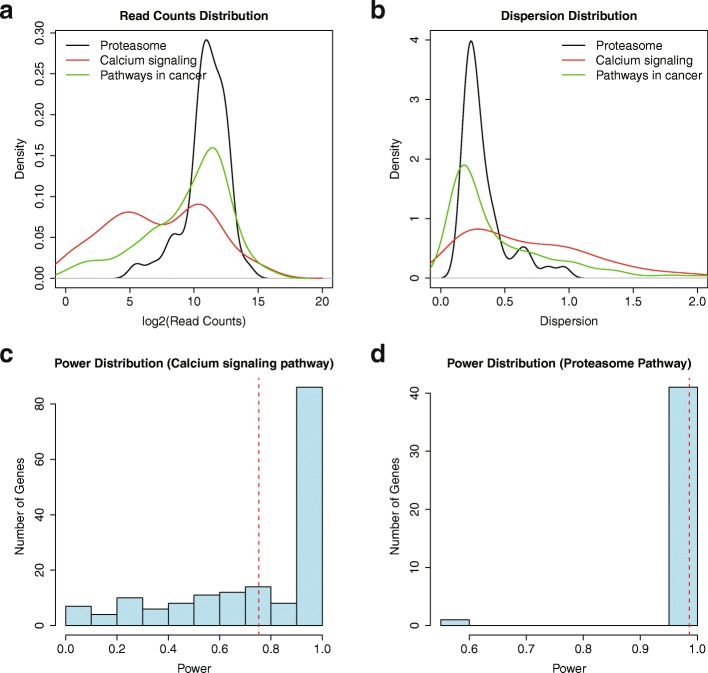
Sample size estimation for interested genes. **a** The read counts distribution for genes in three KEGG pathways in TCGA READ dataset; (**b**) The dispersion distribution for genes in three KEGG pathways in TCGA READ dataset; (**c**) The power distribution based on the count and dispersion distributions in TCGA READ dataset for genes in Calcium signaling pathway when sample size equals 71. The red lines indicate the mean value of power distribution. **d** The power distribution based on the count and dispersion distributions in TCGA READ dataset for genes in Proteasome pathway when sample size equals 71. The red lines indicate the mean value of the power distribution

As illustrated in Fig. 4a and b, genes in Proteasome (KEGG pathway 03050), Calcium Signaling (KEGG pathway 04020) and Pathways in Cancer (KEGG pathway 05200) have distinguishable read counts and a dispersion distribution in TCGA READ dataset. The genes in Proteasome pathway have very high read counts and a low dispersion, whereas genes in Calcium Signaling pathway have low read counts and a high dispersion, which may be a reflection of their functions related to Rectum Adenocarcinoma (Fig. 4c, and d, Additional file 1: Table S4).

Furthermore, we demonstrated that different sample size estimations result in different TCGA datasets with KEGG pathway “Pathways in Cancer” (Additional file 1: Table S5). RnaSeqSampleSize estimated a sample size of 45 for “Pathways in Cancer” genes (Additional file 1: Table S5, in boldface) vs 42 for all genes (Additional file 1: Table S3, in boldface) in READ dataset if we use the same parameters as previously.

### Power curve visualization for different parameters

**Fig. 5:**
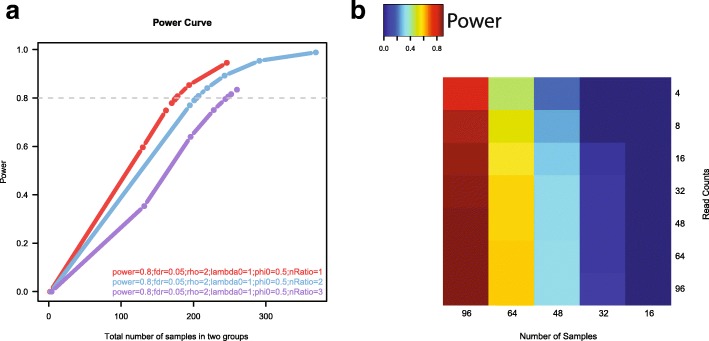
Power curve visualization and parameter optimization by RnaSeqSampleSize. **a** Power curves for balanced (same sample size in two groups) and unbalanced (different sample size in two groups) experiment design. The power curves indicate that the balanced experiment design (red line) will achieve the highest power with the same total number of samples; (**b**) Optimization of parameters in sample size estimation. The dispersion and fold change were set as 0.5 and two, respectively. A power matrix with different pairs of numbers of samples and read counts were generated. The power distribution indicates that the number of samples plays a more significant role in determining the power, and suggests at least 96 samples should be used in RNA-Seq experiments with these parameters to get 0.8 power

Power curves are widely used to analyze and compare sample size estimation results. To demonstrate the power curve visualization feature in RnaSeqSampleSize, we produced three power curves based on different scenarios. As displayed in Fig. 5a, the X-axis indicates the total number of samples used in two groups, and the Y-axis indicates the estimated power. There are three types of sample allocation design: 1:1 sample size in two groups (red curve); 2:1 sample size in two groups (blue curve); 3:1 sample size in two groups (purple curve). The relationship between power and the number of samples can be easily visualized. In the example displayed in Fig. 5a, the power curves indicate that the balanced (sample size 1:1) experiment design (red curve) will achieve the highest power when the same total number of samples is used.

### Parameter optimization for experiment design

The RNA-seq experiment design is often limited by the availability of the budget. The optimization feature in RnaSeqSampleSize can be used to identify the optimal parameters that will achieve the highest power while staying under the budget. To demonstrate the parameter optimization feature, we attempted to optimize numbers of samples and read counts while fixing all other parameters (fold change: 2; dispersion: 1; FDR: 0.05) by generating a power matrix (Fig. 5b). The estimated power was less than 0.1 when 16 samples were used even if the read count was as high as 96. When the number of samples increased to 96, however, the estimated power increased to 0.8, even when the read count was as low as eight. This matrix indicates that the number of samples plays a more significant role in determining the power than the read count, which is consistent with the previous report [20].

## Material and methods

### Software development and data acquisition

RnaSeqSampleSize was developed in R language [21] and compiled into a software package following the guidelines of Bioconductor [22].

The web interface of RnaSeqSampleSize was developed using Shiny package (http://shiny.rstudio.com/) in R language [21].

The TCGA data used as real data examples in RnaSeqSampleSize were downloaded from Genomic Data Commons Data Portal (https://gdc-portal.nci.nih.gov/). The gene expression data of 13 types of cancers were downloaded and included as references in RnaSeqSampleSizeData package in Bioconductor [22].

### Algorithm

In previous research, we have reported a sample size calculation method based the exact test for a single-gene comparison [10]. In this method, we used the concept of pseudo counts [23, 24]. Because the question of interest is to identify the differential gene expression between two groups, the corresponding testing hypothesis is.

H_0_ : γ_0_ = γ_1_vs H_1_ : γ_0_ ≠ γ_1_,

where γ_i_ represents the gene expression level of group i (*i* = 0,1). In order to perform sample size calculations, it is necessary to construct a power function for the testing described above. The power of a test is the probability that the null hypothesis is rejected when the alternative hypothesis is true. For a given marginal type I error level α to reject the null hypothesis, the power can be expressed as

1 $$ \varepsilon \left(n,\rho, {\mu}_{\mathrm{g}},{\upvarphi}_g,\omega, \upalpha \right)=\sum \limits_{y_0=0}^{\infty}\sum \limits_{y_1=0}^{\infty }f\left({n\omega \rho \mu}_g,\frac{\upvarphi_g}{\mathrm{n}}\right)f\left({n\mu}_{\mathrm{g}},\frac{\upvarphi_g}{\mathrm{n}}\right)I\left(\mathrm{p}\left({y}_1,{y}_0\right)<\alpha \right) $$

Where g is the single gene in comparison; *n* is the number of samples in each group; *ρ* is the fold change between the two groups; *μ*_*g*_ is the average read count for gene g in the control group; φ_*g*_ is the dispersion parameter for gene g in the control group; *ω* is the geometric mean of normalization factors between the two groups; α is type I error rate; *y*_1_ and *y*_0_ are pseudo counts [25] in the two groups; *f*(*μ*, φ) is the probability mass function of the negative binomial distribution with mean *μ* as well as dispersion φ; and *I*(p(*y*_1_, *y*_0_) < *α*) denotes the indicator function for the *p* value of the exact test [25].

In reality, thousands of genes are examined in an RNA-seq experiment, and those genes are tested simultaneously for significance of differential expression. In such cases, multiple testing problem should be considered. We proposed a false discovery rate (FDR) controlled method in previous research [10], which is defined as the expected proportion of false discoveries among rejected null hypotheses. In this method, the marginal type I error level α will be adjusted to α∗ to guarantee the expected number of true rejections at a given FDR.

To calculate the sample size, we need to pre-specific the parameters estimated from the differentially expressed genes. However, we may not be able to know or determine which genes were differentially expressed in a real dataset. To deal this issue, we assume the distribution of average read count in control group (*μ*_*g*_ in formula 2) and dispersion (φ_*g*_ in formula 2) for differential expressed genes were the same as all genes. Then we randomly selected genes from the real data set and treated them as differentially expressed. Functions in edgeR package were wrapped and used to estimate the average read counts and dispersion distribution. If M1 was the number of differential expressed genes in the dataset, we randomly selected M1 genes from all genes and used their average read counts and dispersions from the distribution to represent differential genes. As a result, the power of detecting these M1 differential genes can be calculated:

2 $$ \mathrm{Power}=\frac{\sum_{\mathrm{g}=1}^{\mathrm{M}1}\varepsilon \left(n,\rho, {\mu}_{\mathrm{g}},{\upvarphi}_{\mathrm{g}},\omega, \upalpha \ast \right)}{\mathrm{M}1} $$

The value of power in formula (2) is highly dependent on the selected differential genes. When the number of differential expressed genes is small, different genes will be selected in each replication and results in a significant diversity among the power in replications. Motivated by the ensemble method in machine learning, we average all the powers calculated from the replications to obtain a robust estimation of power.

This re-sampling process was repeated several times (1000 by default) to get a power distribution and the power distribution was summarized (averaged by default) to obtain a robust estimation of power. Then, RnaSeqSampleSize package will use the numerical approach to find the n when the robust estimation of power is equal to the desired level.

## Conclusion

Sample size estimation is a critical step in RNA sequencing experimental design. It provides an important solution for balancing the number of samples and the statistical power. Here, we presented the power and sample size estimation software RnaSeqSampleSize to overcome the current limitations and provide a less conservative yet more accurate and reliable result. RnaSeqSampleSize provides more efficient computations compared to previous methods; additionally, it provides several novel visualization and optimization features as well as a much desired graphical user web interface, which allows investigators without a background in programming to easily conduct sample size calculation (Additional file 1: Figure S1).

What separates RnaSeqSampleSize from the other RNA-Seq power analysis tools is its usage of reference data, which can help generate a reliable read count and dispersion distribution. We preloaded the TCGA datasets for users without reference data. The TCGA dataset provides a comprehensive reference for cancer tissues samples, but the reference datasets for non-cancer or non-tissue samples are not currently included. As more and more RNA sequencing datasets become publically available, we will continually update the reference dataset.

## Availability

Home page:

http://www.bioconductor.org/packages/release/bioc/html/RnaSeqSampleSize.html

Web interface:

http://cqs.mc.vanderbilt.edu/shiny/RnaSeqSampleSize/

## Additional file

Additional file 1: **Table S1.** The improvement in efficiency in RnaSeqSampleSize package. **Table S2.** Estimated sample size for RNA-Seq experiments in different cancer types by single parameter method. **Table S3.** Estimated sample size for RNA-Seq experiments in different cancer types by real data distribution based method. For each cancer type, we used the related TCGA dataset to estimate the read count and dispersion distribution. **Table S4.** Estimated sample size for RNA-Seq experiments in different cancer types by real data distribution based method, only the genes in interested KEGG pathway were considered. **Table S5.** Estimated sample size for RNA-Seq experiments in different cancer types by real data distribution based method, only the genes in KEGG pathway ID 05200 (Pathways in Cancer) were considered. **Figure S1.** A screen shot of user interface of RnaSeqSampleSize package. (DOCX 217 kb)

## References

[1] Wang Z, Gerstein M, Snyder M (2009). RNA-Seq: a revolutionary tool for transcriptomics. Nat Rev Genet.

[2] Jung SH, Bang H, Young S (2005). Sample size calculation for multiple testing in microarray data analysis. Biostatistics.

[3] Müller P, Parmigiani G, Robert C, Rousseau J (2004). Optimal sample size for multiple testing: the case of gene expression microarrays. J Am Stat Assoc.

[4] Busby MA, Stewart C, Miller CA, Grzeda KR, Marth GT (2013). Scotty: a web tool for designing RNA-Seq experiments to measure differential gene expression. Bioinformatics.

[5] Chen Z, Liu J, Ng HK, Nadarajah S, Kaufman HL, Yang JY, Deng Y (2011). Statistical methods on detecting differentially expressed genes for RNA-seq data. BMC Syst Biol.

[6] Fang Z, Cui X (2011). Design and validation issues in RNA-seq experiments. Brief Bioinform.

[7] Robinson MD, Oshlack A (2010). A scaling normalization method for differential expression analysis of RNA-seq data. Genome Biol.

[8] Anders S, Huber W (2010). Differential expression analysis for sequence count data. Genome Biol.

[9] Hart SN, Therneau TM, Zhang Y, Poland GA, Kocher JP (2013). Calculating sample size estimates for RNA sequencing data. J Comput Biol.

[10] Li CI, Su PF, Shyr Y (2013). Sample size calculation based on exact test for assessing differential expression analysis in RNA-seq data. BMC bioinformatics.

[11] Liu Y, Zhou J, White KP (2014). RNA-seq differential expression studies: more sequence or more replication?. Bioinformatics.

[12] Ching T, Huang S, Garmire LX (2014). Power analysis and sample size estimation for RNA-Seq differential expression. RNA.

[13] Li CI, Samuels DC, Zhao YY, Shyr Y, Guo Y. Power and sample size calculations for high-throughput sequencing-based experiments. Brief Bioinform. 2017; https://www.ncbi.nlm.nih.gov/pubmed/28605403. 10.1093/bib/bbx061 PMC6291796 28605403

[14] Therneau TM, Hart SN, Kocher JP. RNASeqPower: Calculating samples Size estimates for RNA Seq studies. R package version 1.18.0. 2013.

[15] Guo Y, Li J, Li CI, Shyr Y, Samuels DC (2013). MitoSeek: extracting mitochondria information and performing high-throughput mitochondria sequencing analysis. Bioinformatics.

[16] Wu H, Wang C, Wu ZJ (2015). PROPER: comprehensive power evaluation for differential expression using RNA-seq. Bioinformatics.

[17] Zhou X, Lindsay H, Robinson MD (2014). Robustly detecting differential expression in RNA sequencing data using observation weights. Nucleic Acids Res.

[18] Yu L, Fernandez S, Brock G (2017). Power analysis for RNA-Seq differential expression studies. BMC Bioinformatics.

[19] Croft D, O’Kelly G, Wu G, Haw R, Gillespie M, Matthews L, Caudy M, Garapati P, Gopinath G, Jassal B (2011). Reactome: a database of reactions, pathways and biological processes. Nucleic Acids Res.

[20] Rapaport F, Khanin R, Liang Y, Pirun M, Krek A, Zumbo P, Mason CE, Socci ND, Betel D (2013). Comprehensive evaluation of differential gene expression analysis methods for RNA-seq data. Genome Biol.

[21] R Core Team (2016). R: a language and environment for statistical computing. R foundation for statistical computing.

[22] Huber W, Carey VJ, Gentleman R, Anders S, Carlson M, Carvalho BS, Bravo HC, Davis S, Gatto L, Girke T (2015). Orchestrating high-throughput genomic analysis with Bioconductor. Nat Methods.

[23] Robinson MD, Smyth GK (2008). Small-sample estimation of negative binomial dispersion, with applications to SAGE data. Biostatistics.

[24] Robinson MD, Smyth GK (2007). Moderated statistical tests for assessing differences in tag abundance. Bioinformatics.

[25] Robinson MD, McCarthy DJ, Smyth GK (2010). edgeR: a Bioconductor package for differential expression analysis of digital gene expression data. Bioinformatics.

