# Supplementary material for: RnaSeqSampleSize: real data based sample size estimation for RNA sequencing
Source: BMC Bioinformatics. 2018 May 30;19:191. doi: 10.1186/s12859-018-2191-5 (PMC5975570; doi:10.1186/s12859-018-2191-5)
Supplement: Supplementary file 1 — Table S1. The improvement in efficiency in RnaSeqSampleSize package. Table S2. Estimated sample size for RNA-Seq experiments in different cancer types by single parameter method. Table S3. Estimated sample size for RNA-Seq experiments in different cancer types by real data distribution based method. For each cancer type, we used the related TCGA dataset to estimate the read count and dispersion distribution. Table S4. Estimated sample size for RNA-Seq experiments in different cancer types by real data distribution based method, only the genes in interested KEGG pathway were considered. Table S5. Estimated sample size for RNA-Seq experiments in different cancer types by real data distribution based method, only the genes in KEGG pathway ID 05200 (Pathways in Cancer) were considered. Figure S1. A screen shot of user interface of RnaSeqSampleSize package. (DOCX 217 kb) [file 12859_2018_2191_MOESM1_ESM.docx]

**Supplementary Tables:**

sTable 1: The improvement in efficiency in RnaSeqSampleSize package. We used the following parameters as an example to demonstrate the improvement. The NAs indicated that the previous method can’t be used with these parameters.

| **Read counts** | **Dispersion** | **Fold change** | **Power** | **FDR** | **Time (previous)** | **Time (RnaSeqSampleSize)** |
| --- | --- | --- | --- | --- | --- | --- |
| 1 | 0.1 | 2 | 0.8 | 0.05 | 10 min | 0.2 sec |
| 5 | 0.5 | 2 | 0.8 | 0.05 | 40 min | 1.7 sec |
| 10 | 0.5 | 4 | 0.8 | 0.01 | 2 hour | 5.1 sec |
| 20 | 1 | 2 | 0.9 | 0.01 | NA | 14.6 sec |
| 50 | 1 | 2 | 0.9 | 0.1 | NA | 14.8 sec |
| 500 | 2 | 3 | 0.9 | 0.1 | NA | 34.9 sec |

sTable 2: Estimated sample size for RNA-Seq experiments in different cancer types by single parameter method. For each cancer type, we used the 95% quantile of gene dispersions from related TCGA dataset as its maximal dispersion, to avoid the influence of outliers. The dispersion was labeled in the bracket after the cancer type.

| Parameters | | | | Cancer Types | | | | | | | | | | | | |
| --- | --- | --- | --- | --- | --- | --- | --- | --- | --- | --- | --- | --- | --- | --- | --- | --- |
| Power | FDR | Fold Change | Min Count | BLCA  (3.4) | BRCA  (3.0) | CESC  (3.5) | COAD  (2.6) | HNSC  (2.7) | KIRC  (2.1) | LGG  (1.9) | LUAD  (2.9) | LUSC  (2.8) | PRAD  (1.9) | **READ**  **(2.0)** | THCA  (2.2) | UCEC  (3.3) |
| **0.8** | 0.01 | 1.5 | 1 | 1184 | 1074 | 1212 | 962 | 990 | 822 | 766 | 1046 | 1018 | 766 | 794 | 850 | 1156 |
|  |  |  | 10 | 976 | 864 | 1005 | 752 | 780 | 612 | 556 | 836 | 808 | 556 | 584 | 640 | 948 |
|  |  | 2 | 1 | 400 | 361 | 410 | 323 | 332 | 274 | 255 | 352 | 342 | 255 | 264 | 284 | 390 |
|  |  |  | 10 | 336 | 298 | 346 | 259 | 269 | 211 | 191 | 288 | 278 | 191 | 201 | 220 | 327 |
|  | **0.05** | 1.5 | 1 | 990 | 896 | 1013 | 803 | 826 | 686 | 639 | 873 | 850 | 639 | 663 | 709 | 967 |
|  |  |  | 10 | 814 | 721 | 838 | 627 | 651 | 511 | 464 | 698 | 674 | 464 | 487 | 534 | 791 |
|  |  | **2** | 1 | 334 | 302 | 342 | 269 | 277 | 229 | 213 | 294 | 286 | 213 | 221 | 237 | 326 |
|  |  |  | **10** | 281 | 248 | 289 | 216 | 224 | 176 | 160 | 240 | 232 | 160 | **168** | 184 | 273 |
| 0.9 | 0.01 | 1.5 | 1 | 1398 | 1265 | 1431 | 1133 | 1166 | 969 | 903 | 1232 | 1199 | 903 | 936 | 1003 | 1365 |
|  |  |  | 10 | 1153 | 1020 | 1186 | 888 | 921 | 722 | 656 | 987 | 954 | 656 | 689 | 756 | 1119 |
|  |  | 2 | 1 | 471 | 425 | 483 | 380 | 391 | 323 | 300 | 414 | 403 | 300 | 311 | 334 | 460 |
|  |  |  | 10 | 396 | 351 | 408 | 305 | 316 | 248 | 225 | 339 | 328 | 225 | 237 | 259 | 385 |
|  | 0.05 | 1.5 | 1 | 1183 | 1072 | 1211 | 960 | 988 | 820 | 764 | 1044 | 1016 | 764 | 792 | 848 | 1155 |
|  |  |  | 10 | 975 | 863 | 1003 | 751 | 779 | 611 | 555 | 835 | 807 | 555 | 583 | 639 | 947 |
|  |  | 2 | 1 | 399 | 360 | 408 | 322 | 331 | 273 | 254 | 350 | 341 | 254 | 264 | 283 | 389 |
|  |  |  | 10 | 335 | 297 | 345 | 258 | 268 | 210 | 191 | 287 | 277 | 191 | 200 | 219 | 326 |

sTable 3: Estimated sample size for RNA-Seq experiments in different cancer types by real data distribution based method. For each cancer type, we used the related TCGA dataset to estimate the read count and dispersion distribution. Other parameters were the same with sTable 2. As a result, we can find that the estimated sample size by real data distribution based method was much lesser than single parameter method.

| Parameters | | | | Cancer Types | | | | | | | | | | | | |
| --- | --- | --- | --- | --- | --- | --- | --- | --- | --- | --- | --- | --- | --- | --- | --- | --- |
| Power | FDR | Fold Change | Library Size | BLCA | BRCA | CESC | COAD | HNSC | KIRC | LGG | LUAD | LUSC | PRAD | **READ** | THCA | UCEC |
| **0.8** | 0.01 | 1.5 | 10^7^ | 285 | 245 | 217 | 306 | 232 | 140 | 161 | 210 | 236 | 169 | 159 | 182 | 279 |
|  |  |  | 10^8^ | 270 | 230 | 202 | 290 | 216 | 128 | 151 | 197 | 225 | 157 | 147 | 166 | 266 |
|  |  | 2 | 10^7^ | 99 | 85 | 75 | 106 | 80 | 48 | 56 | 73 | 82 | 58 | 55 | 63 | 97 |
|  |  |  | 10^8^ | 94 | 80 | 71 | 101 | 75 | 45 | 53 | 69 | 79 | 55 | 51 | 58 | 92 |
|  | **0.05** | 1.5 | 10^7^ | 230 | 198 | 174 | 246 | 188 | 113 | 130 | 170 | 191 | 137 | 128 | 146 | 226 |
|  |  |  | 10^8^ | 219 | 186 | 163 | 234 | 175 | 104 | 122 | 160 | 182 | 127 | 119 | 134 | 215 |
|  |  | **2** | 10^7^ | 80 | 69 | 61 | 86 | 65 | 39 | 45 | 59 | 66 | 47 | 44 | 51 | 78 |
|  |  |  | **10^8^** | 76 | 65 | 57 | 82 | 61 | 36 | 43 | 56 | 64 | 44 | **42** | 47 | 75 |
| 0.9 | 0.01 | 1.5 | 10^7^ | 472 | 426 | 374 | 514 | 393 | 249 | 268 | 363 | 391 | 274 | 275 | 309 | 452 |
|  |  |  | 10^8^ | 446 | 389 | 344 | 483 | 358 | 218 | 243 | 334 | 371 | 248 | 249 | 276 | 424 |
|  |  | 2 | 10^7^ | 163 | 147 | 129 | 177 | 135 | 85 | 92 | 125 | 135 | 94 | 95 | 106 | 156 |
|  |  |  | 10^8^ | 155 | 135 | 119 | 168 | 124 | 76 | 84 | 116 | 129 | 86 | 87 | 96 | 147 |
|  | 0.05 | 1.5 | 10^7^ | 384 | 346 | 304 | 418 | 320 | 202 | 218 | 295 | 318 | 224 | 224 | 252 | 369 |
|  |  |  | 10^8^ | 363 | 316 | 280 | 393 | 292 | 177 | 198 | 272 | 302 | 203 | 203 | 225 | 346 |
|  |  | 2 | 10^7^ | 133 | 119 | 105 | 144 | 110 | 69 | 75 | 102 | 110 | 77 | 77 | 87 | 127 |
|  |  |  | 10^8^ | 126 | 110 | 97 | 136 | 101 | 62 | 69 | 94 | 105 | 71 | 71 | 78 | 120 |

sTable 4: Estimated sample size for RNA-Seq experiments in different cancer types by real data distribution based method, only the genes in interested KEGG pathway were considered. For each pathway, we used genes in TCGA READ dataset to estimate the read count and dispersion distribution. Other parameters were the same with sTable 2.

| Parameters | | | | Cancer Types: READ | | | |
| --- | --- | --- | --- | --- | --- | --- | --- |
| Power | FDR | Fold Change | Library Size | Citrate cycle  (00020) | Proteasome  (03050) | Pathways In Cancer  (05200) | Insulin Secretion  (04911) |
| **0.8** | 0.01 | 1.5 | 10^7^ | 56 | 45 | 171 | 254 |
|  |  |  | 10^8^ | 56 | 45 | 166 | 244 |
|  |  | 2 | 10^7^ | 19 | 16 | 76 | 88 |
|  |  |  | 10^8^ | 19 | 16 | 72 | 84 |
|  | **0.05** | 1.5 | 10^7^ | 46 | 37 | 67 | 206 |
|  |  |  | 10^8^ | 46 | 37 | 65 | 198 |
|  |  | **2** | 10^7^ | 16 | 13 | 41 | 71 |
|  |  |  | **10^8^** | 16 | 13 | 39 | 68 |
| 0.9 | 0.01 | 1.5 | 10^7^ | 80 | 63 | 192 | 375 |
|  |  |  | 10^8^ | 80 | 63 | 186 | 364 |
|  |  | 2 | 10^7^ | 28 | 22 | 99 | 129 |
|  |  |  | 10^8^ | 28 | 22 | 92 | 124 |
|  | 0.05 | 1.5 | 10^7^ | 67 | 52 | 87 | 307 |
|  |  |  | 10^8^ | 67 | 52 | 81 | 298 |
|  |  | 2 | 10^7^ | 23 | 18 | 50 | 106 |
|  |  |  | 10^8^ | 23 | 18 | 46 | 102 |

sTable 5: Estimated sample size for RNA-Seq experiments in different cancer types by real data distribution based method, only the genes in KEGG pathway ID 05200 (Pathways in Cancer) were considered. For each cancer type, we used genes of “Pathways in Cancer” in the related TCGA dataset to estimate the read count and dispersion distribution. Other parameters were the same with sTable 2.

| Parameters | | | | Cancer Types | | | | | | | | | | | | |
| --- | --- | --- | --- | --- | --- | --- | --- | --- | --- | --- | --- | --- | --- | --- | --- | --- |
| Power | FDR | Fold Change | Library Size | BLCA | BRCA | CESC | COAD | HNSC | KIRC | LGG | LUAD | LUSC | PRAD | **READ** | THCA | UCEC |
| **0.8** | 0.01 | 1.5 | 10^7^ | 266 | 214 | 228 | 264 | 199 | 131 | 163 | 166 | 203 | 158 | 164 | 178 | 243 |
|  |  |  | 10^8^ | 270 | 215 | 218 | 265 | 206 | 129 | 159 | 166 | 198 | 153 | 160 | 178 | 244 |
|  |  | 2 | 10^7^ | 92 | 74 | 79 | 92 | 69 | 45 | 57 | 58 | 70 | 55 | 57 | 62 | 84 |
|  |  |  | 10^8^ | 94 | 75 | 76 | 92 | 72 | 45 | 55 | 58 | 69 | 53 | 56 | 62 | 85 |
|  | **0.05** | 1.5 | 10^7^ | 215 | 174 | 184 | 213 | 161 | 106 | 133 | 135 | 165 | 128 | 132 | 144 | 197 |
|  |  |  | 10^8^ | 218 | 174 | 176 | 214 | 167 | 104 | 129 | 135 | 161 | 124 | 129 | 144 | 198 |
|  |  | **2** | 10^7^ | 75 | 60 | 64 | 74 | 56 | 37 | 46 | 47 | 57 | 44 | 46 | 50 | 69 |
|  |  |  | **10^8^** | 76 | 61 | 61 | 75 | 58 | 36 | 45 | 47 | 56 | 43 | **45** | 50 | 69 |
| 0.9 | 0.01 | 1.5 | 10^7^ | 420 | 344 | 381 | 443 | 314 | 240 | 261 | 272 | 313 | 242 | 282 | 300 | 378 |
|  |  |  | 10^8^ | 419 | 339 | 351 | 439 | 326 | 227 | 245 | 272 | 300 | 229 | 261 | 295 | 380 |
|  |  | 2 | 10^7^ | 145 | 118 | 131 | 153 | 108 | 83 | 90 | 94 | 108 | 83 | 97 | 103 | 130 |
|  |  |  | 10^8^ | 145 | 117 | 121 | 152 | 113 | 79 | 85 | 95 | 104 | 79 | 90 | 102 | 131 |
|  | 0.05 | 1.5 | 10^7^ | 342 | 281 | 310 | 361 | 257 | 194 | 214 | 222 | 257 | 199 | 229 | 244 | 310 |
|  |  |  | 10^8^ | 343 | 277 | 286 | 358 | 266 | 184 | 201 | 222 | 247 | 188 | 213 | 240 | 311 |
|  |  | 2 | 10^7^ | 118 | 97 | 107 | 125 | 89 | 67 | 74 | 77 | 89 | 68 | 79 | 84 | 107 |
|  |  |  | 10^8^ | 119 | 96 | 99 | 124 | 92 | 64 | 70 | 77 | 86 | 65 | 74 | 83 | 108 |

**Supplementary Figures:**


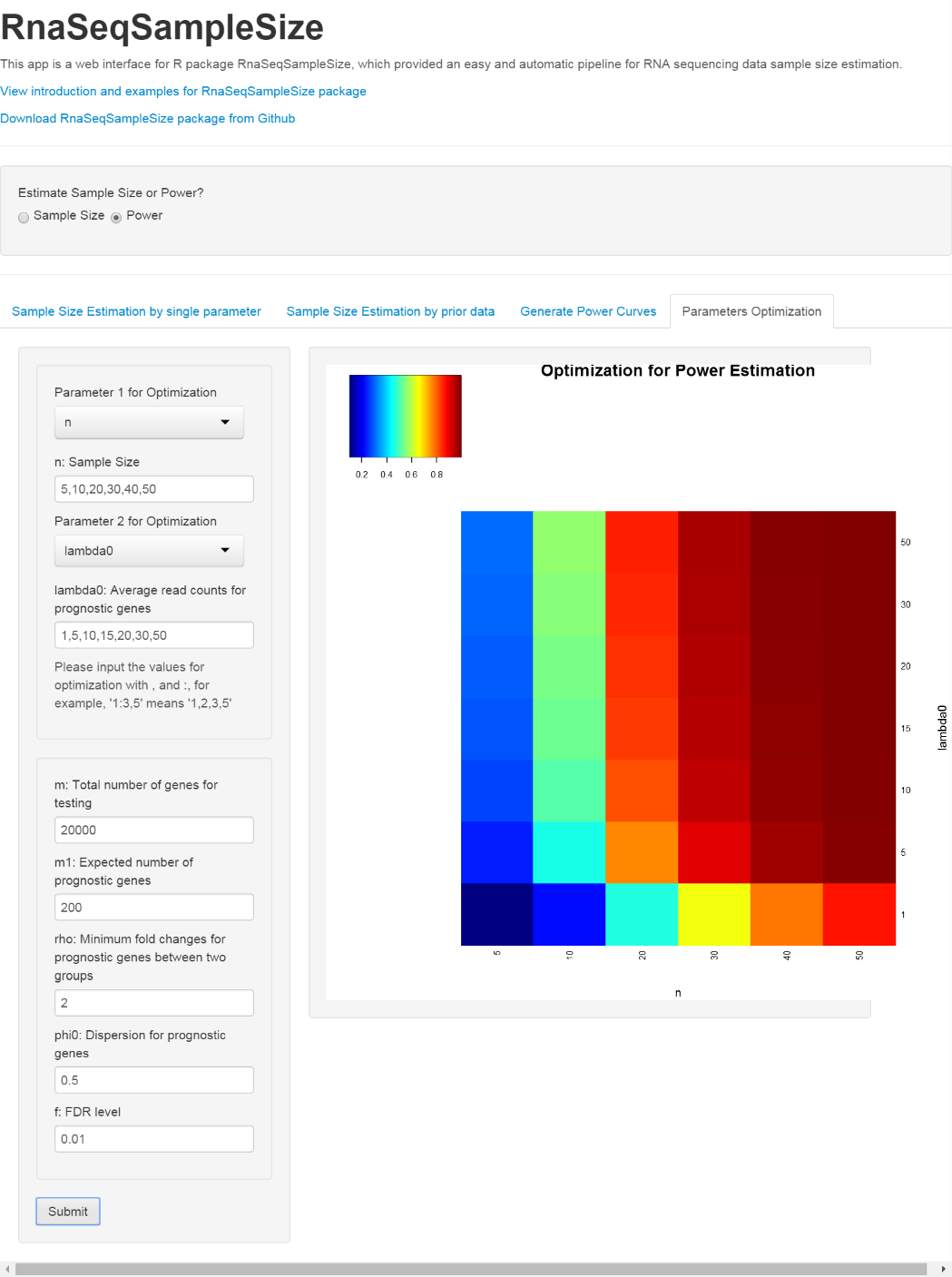


sFigure 1: A screen shot of user interface of RnaSeqSampleSize package.
